# Supplementary material for: Proteomic Profiling Identifies Specific Leukemic Stem Cell-Associated Protein Expression Patterns in Pediatric AML Patients
Source: Cancers (Basel). 2022 Jul 22;14(15):3567. doi: 10.3390/cancers14153567 (PMC9332109; doi:10.3390/cancers14153567)
Supplement: Supplementary file 1 [file cancers-14-03567-s001.zip › Supplementary Figure legends_Petersen et al.pdf]

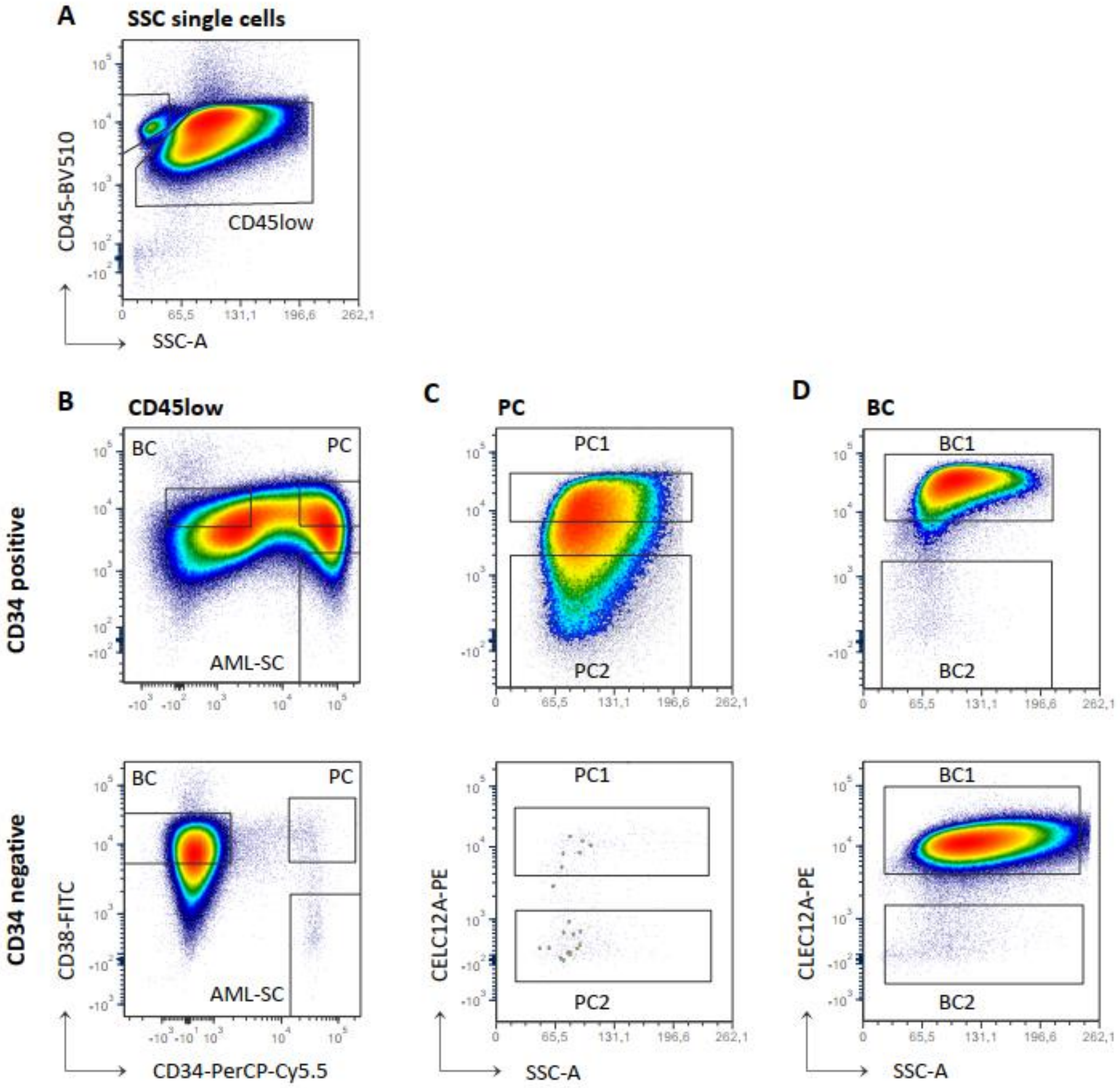

Figure S1. Gating strategy for Fluorescence-activated cell sorting of acute myeloid leukemia cell subsets. (A) Initially, live, SSC (side scatter) single cells were selected by exclusion of dead cells, debris, and doublets (not shown). Next, the leukemic blasts were gated as CD45low, accounting for 94.9–96.5% of the SSC single cells in the five patient samples. (B) The AML-SC, PC, and BC were gated within the CD45low cells as CD34+CD38–, CD34+CD38+, and CD34–CD38+, respectively. The (C) PC and (D) BC subsets were further sorted as either CLEC12A+ or CLEC12A–, denoted by the suffixes 1 and 2, respectively. As CD34 expression levels varied between the AML samples, representative CD34-positive (middle panel) and CD34-negative (lower panel) samples are shown.

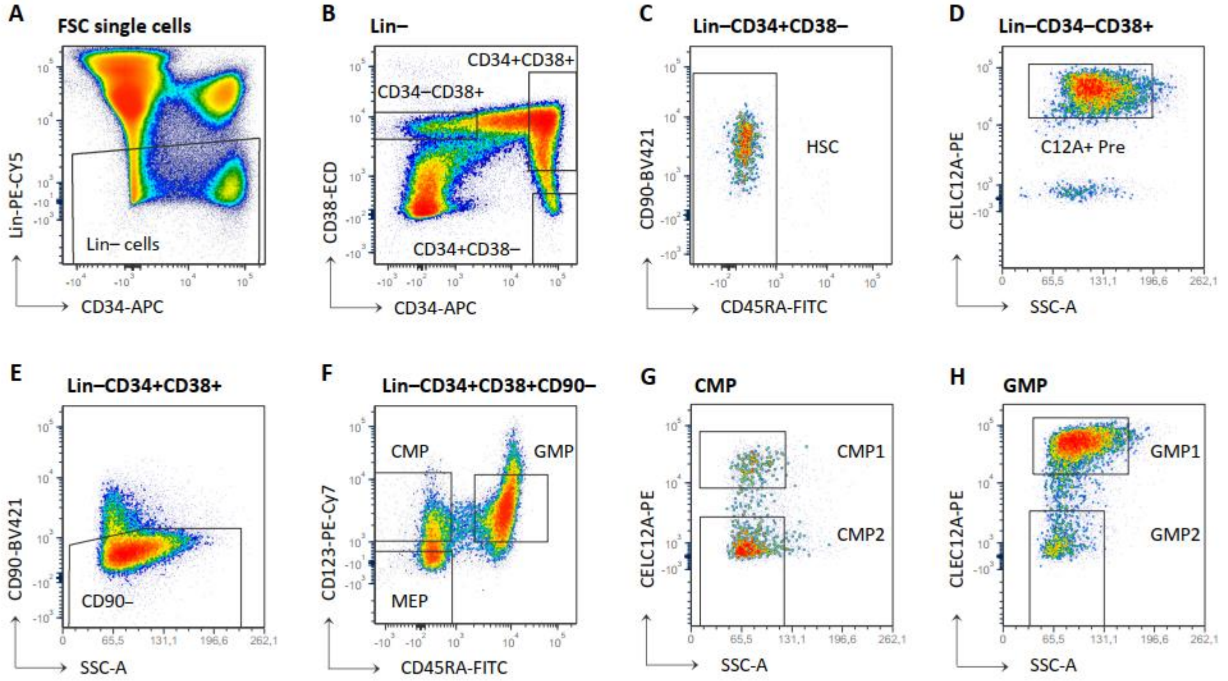

Figure S2. Gating strategy for F**luorescence-activated cell** *s***orting** of immature cell subsets from hematologically healthy controls. (A) After exclusion of dead cells, debris, and doublets (not shown), Lin– cells were selected from the FSC single cells. (B) Within the Lin– cells, CD34+CD38–, CD34+CD38+, and CD34–CD38+ cell subsets were gated. (C) From the CD34+CD38– cells, the HSCs were gated as Lin–CD34+CD38–CD45RA–CD90+/–. (D) The CLEC12A+ Pre subset was defined within the Lin–CD34–CD38+ cells (defined in (B)). (E) Of the Lin–CD34+CD38+ progenitor compartment (defined in [B]), the CD90– cells were selected in an SSC area vs. CD90 plot. (F) To further subdivide the progenitors, the Lin–CD34+CD38+CD90– cells were gated in a CD123 vs. CD45RA plot, whereby the MEP, GMP, and CMP compartments could be defined. The (G) CMP and (H) GMP were further differentiated according to CLEC12A expression, where the suffixes 1 and 2 denote CLEC12A positivity and negativity, respectively.

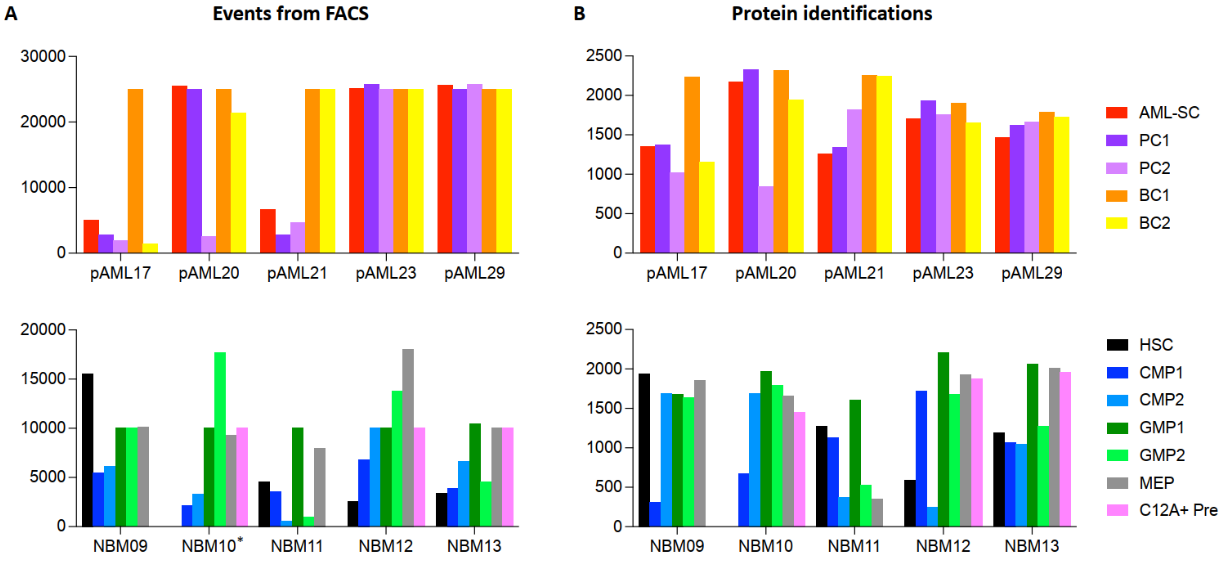

Figure S3. Output from Fluorescence-activated cell sorting and mass spectrometry. (A) Number of FACS-sorted events in each of the 57 samples. (B) Number of protein identifications in each of the 57 samples. Eight samples (one PC2, one HSC, two CMP1, two CMP2, one GMP2, and one MEP) with less than 1000 protein identifications were excluded from further analyses. Upper panels: AML samples (pAML). Lower panels: Hematologically healthy controls (NBM). *FACS sorting of BM samples from NBM10 was done twice, and the sorted CMP1, CMP2, GMP2, and MEP subsets pooled prior to MS analysis.

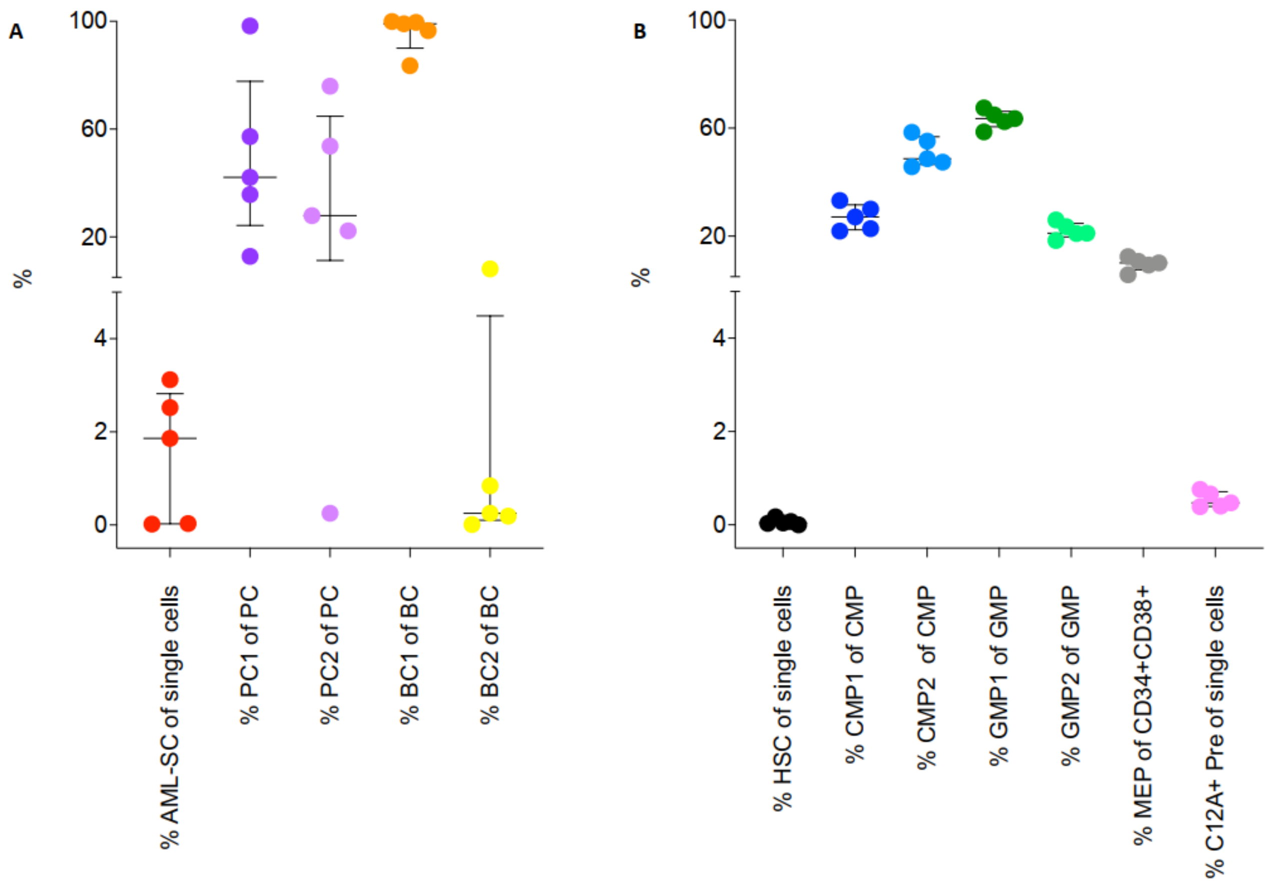

Figure S4. Distribution of Fluorescence-activated cell sorting sorted cell populations. (A) Within the patients, AML-SC represented a low fraction of the live, single cells (median: 1.87%; range: 0.02–3.12). Of the CD34–CD38+ BCs, the vast majority expressed CLEC12A, represented by the BC1 subset (median: 99.09%; range: 83.54–99.94%), whereas the infrequent BC2 subset only represented 0.25% (range: 0.01–8.14%). Interpatient variation was seen within the CD34+CD38+ PC subsets, where the PC1 subset constituted 42.14% (range: 12.78–98.30%), and the PC2 subset 27.88% (range: 0.25–75.93). (B) In the hematologically healthy controls, the HSCs comprised a minute fraction of live, single cells (median: 0.04%; range: 0.00–0.17%), and in one patient (NBM10), HSCs could not be identified in the BM sample. Of the CMPs, a median frequency of 27.10% (range: 21.90– 33.20%) and 48.66% (range: 45.69–58.42%) was found for CMP1 and CMP2, respectively. Within the GMPs, the GMP1 and GMP2 subsets constituted a median of 63.50% (range: 58.60–67.50) and 21.10% (18.40–26.00%), respectively. The MEPs represented 10.10% of the single cells (range: 5.70–12.40%); the CD34–CD38+CLEC12A+ precursors (C12A+ Pre), 0.47% (range: 0.39–0.76%). The sorting gates defining the FACS sorted cell populations are demonstrated in Figs. S1 and S2 for the AML samples and hematologically healthy controls, respectively.

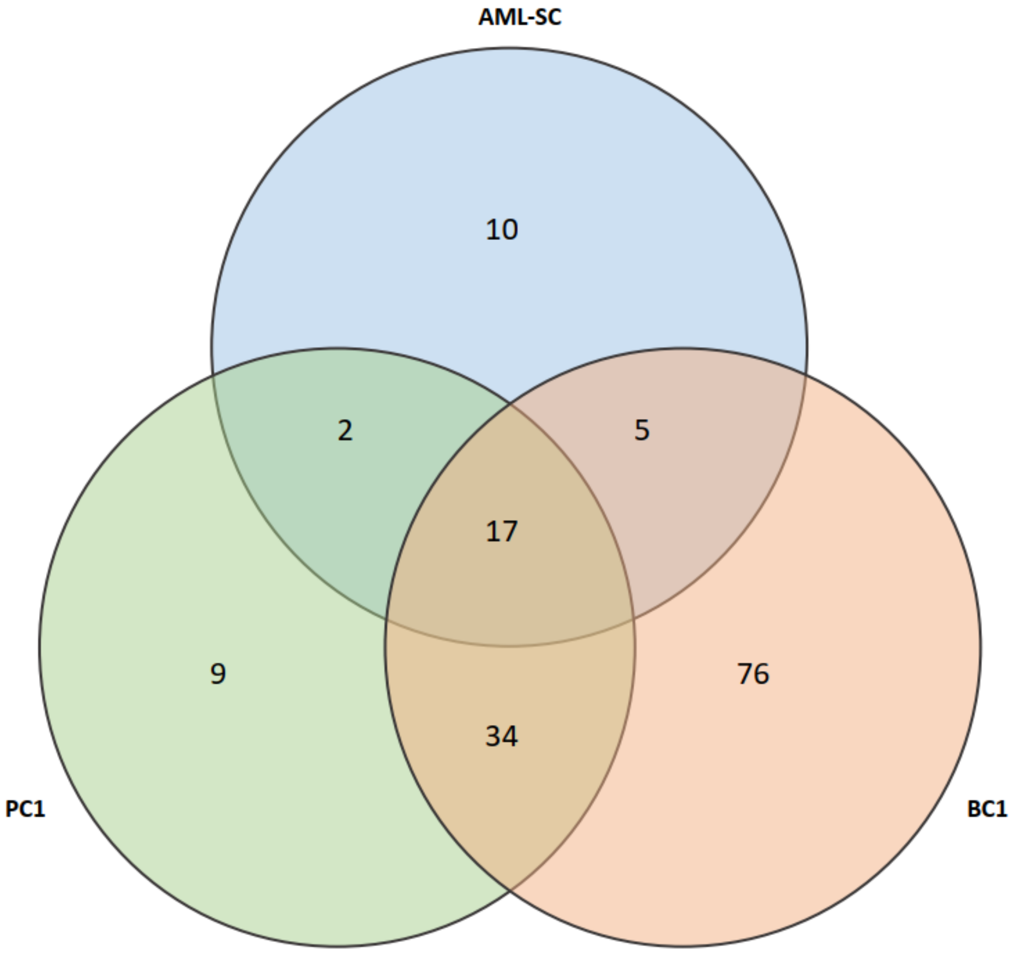

Figure S5. Circle plot of shared and unique protein expressed in leukemic subsets compared with healthy stem cells. The number of proteins shared in the individual comparison analysis relative to HSCs is depicted in the overlapping circles, and the number of unique proteins only present in the individual analyses is depicted in single circles. The 17 proteins shared between all comparative analyses and the 10 proteins unique to AML-SCs are listed in Tables S7 and S8, respectively.
