# Supplementary material for: Proteomic Profiling Identifies Specific Leukemic Stem Cell-Associated Protein Expression Patterns in Pediatric AML Patients
Source: Cancers (Basel). 2022 Jul 22;14(15):3567. doi: 10.3390/cancers14153567 (PMC9332109; doi:10.3390/cancers14153567)
Supplement: Supplementary file 1 [file cancers-14-03567-s001.zip › Supplementary Information_Petersen et al.pdf]

1. Supplementary Methods

1.1. Patients and materials

The mononuclear cells (MNCs) were isolated from the bone marrow (BM) samples of the patients by density gradient separation using Lymphoprep™ (Alere Technologies/Abbott, Kista, Sweden) (centrifugation at 900 g, 30 min), followed by lysis of red blood cells. For the leukemic samples, cell fractions were isolated and harvested according to routine protocol using LeucoSEP™ tubes (Greiner Bio-One™, Fisher Scientific, Roskilde, Denmark) by decanting the supernatant above the porous barrier; for the control samples, the MNC layer was harvested using a Pasteur pipette. Samples were cryopreserved in 20% fetal bovine serum (FBS), (Biowest, Nuaillé, France) with 10% dimethyl sulfoxide (Sigma-Aldrich/Merck, St. Louis, MO, USA) and stored in liquid nitrogen until time of analysis.

1.2. Fluorescence-activated cell sorting (FACS)

Vials of cryopreserved BM MNCs were thawed in a 37°C water bath, transferred to 37°C FBS containing medium, and centrifuged at 250 g for 10 min. After resuspension, the cells were treated with DNAse1 and $MgCL_2$ (both from Sigma-Aldrich/Merck) to prevent cell clumping, followed by staining with the pre-titrated reagents listed in Table S1. For compensation, single-stained beads (for the antibodies, UltraComp eBeads™, Thermo Fisher Scientific, Waltham, MA, USA) and cells (for the viability marker) were used as controls. Samples were sorted on a BD FACSAria III (BD Biosciences, San Jose, CA, USA). The gating strategies for the AML samples and the controls are provided in Figs. S1 and S2, respectively. Gating boundaries were defined using a combination of FMO controls (CD38, CD90, CD45RA, CD123), the Lin+CD34+ population (for CLEC12A), and natural break in fluorescence intensity (for CD34). A total of 57 samples were sorted by FACS (Fig. S3A). In all experiments, cell populations were sorted into 50 μL proteomics lysis buffer (LYSE solution from the in-stage Tip method (PreOmics, Planegg-Martinsried, Germany), placed on dry ice, and stored at −80°C until further preparation. Purity analyses are provided in Table S2. The frequency of the various subsets is shown in Fig. S4.

1.3. Proteomics

The 57 samples sorted by FACS were prepared as described by the manufacturer and finally dissolved in 20 μL LC-LOAD solution (in-stage Tip method (PreOmics, Planegg-Martinsried, Germany) (1). From each preparation, 6 μL was injected in triplicate, giving 171 injections. Generally, the technical replicates were injected at intervals of several days. Mass spectrometry was performed on an Orbitrap Fusion Tribrid mass spectrometer coupled to a Dionex UltiMate™ 3000 RSLC nano system through an EasySpray™ ion source (Thermo Fisher Scientific Instruments, Waltham, MA, USA). Label-free quantitative nano liquid chromatography–tandem mass spectrometry (LFQ nLC-MS/MS) was performed using the universal methods settings, essentially as previously described (2). The 171 raw files were entered into MaxQuant v1.6.6.0 (3) for LFQ analysis using the reviewed UniProt *Homo Sapiens* database downloaded on February 9, 2020. The false discovery rates for peptide spectral matches (PSM) at the protein level and for the site decoy fraction were each set at 1%. The LFQ minimum ratio count was set at 1 and MS/MS was required for LFQ comparisons. As fixed modification, carbamidomethyl (C) was used. For

protein modifications, we used unique and razor peptides, unmodified and modified with oxidation (M) or acetyl (protein N-terminal). The match between runs function was activated and revert sequences were used for decoy search. The protein groups file was entered into Perseus v1.6.14.0 (4) for further processing. The data were filtered for reverse hits, those only identified by site, and for contaminants. Two unique peptides were required for each protein identification. The LFQ values were Log_2 transformed and the average of the triplicates was used. Data were further filtered, removing samples with less than 1000 protein identifications. Thus, 8 of the 57 samples were removed due to a very low protein content, resulting in 49 samples (Fig. S3B). A total of 3123 proteins were identified in the combined sample set, with each sample containing between 1026 and 2328 protein identifications. We finally excluded the proteins that were not identified in all samples, resulting in identification of 456 proteins in 49 samples (Table S3). The median technical coefficient of variation of the protein LFQ values ranged between 6.57% and 18.5% in the 49 samples analyzed, with a median technical coefficient of variation of 11.0%. As CLE12A is a common leukemia-associated antigen and also a promising treatment target, we focused our analyzes of the leukemic blasts on the CLEC12A+ PC1 and BC1 subgroups. However, CLEC12A was either absent or only present on a small fraction of the AML-SCs (data not shown); thus, segregation of AML-SCs based on CLEC12A expression was not performed. Thus, after exclusion of PC2 and BC2, 40 samples with 456 shared proteins were included in the analyses.

1.4. Bioinformatics

Data were analyzed through the use of IPA (QIAGEN Inc., https://www.qiagenbioinformatics.com/products/ingenuity-pathway-analysis). The algorithms developed for use in IPA are described by Krämer et al. (5). In all, 456 proteins were entered and 452 proteins were recognized for analysis.

References

1. Kulak NA, Pichler G, Paron I, Nagaraj N, Mann M. Minimal, encapsulated proteomic-sample processing applied to copy-number estimation in eukaryotic cells. Nat Methods [Internet]. 2014 Mar 2;11(3):319–24. Available from: http://www.nature.com/articles/nmeth.2834
2. Ludvigsen M, Thorlacius-Ussing L, Vorum H, Moyer MP, Stender MT, Thorlacius-Ussing O, et al. Proteomic characterization of colorectal cancer cells versus normal-derived colon mucosa cells: Approaching identification of novel diagnostic protein biomarkers in colorectal cancer. Int J Mol Sci [Internet]. 2020 May 14;21(10):3466. Available from: https://www.mdpi.com/1422-0067/21/10/3466
3. Tyanova S, Temu T, Cox J. The MaxQuant computational platform for mass spectrometry-based shotgun proteomics. Nat Protoc [Internet]. 2016 Dec 27;11(12):2301–19. Available from: http://www.nature.com/articles/nprot.2016.136
4. Tyanova S, Temu T, Sinitcyn P, Carlson A, Hein MY, Geiger T, et al. The Perseus computational platform for comprehensive analysis of (prote)omics data. Nat Methods [Internet]. 2016 Sep 27;13(9):731–40. Available from: http://www.nature.com/articles/nmeth.3901
5. Krämer A, Green J, Pollard J, Tugendreich S. Causal analysis approaches in Ingenuity Pathway Analysis. Bioinformatics [Internet]. 2014 Feb 15;30(4):523–30. Available from: https://academic.oup.com/bioinformatics/article-lookup/doi/10.1093/bioinformatics/btt703
