# Supplementary material for: Proteomic Profiling Identifies Specific Leukemic Stem Cell-Associated Protein Expression Patterns in Pediatric AML Patients
Source: Cancers (Basel). 2022 Jul 22;14(15):3567. doi: 10.3390/cancers14153567 (PMC9332109; doi:10.3390/cancers14153567)
Supplement: Supplementary file 1 [file cancers-14-03567-s001.zip › Table S1_Petersen et al.pdf]

Table S1. Reagents used in FACS experiments

Sample	Specificity	Fluorochrome	MoAb Clone	Catalog #	Company
NBM	CD90	BV421	5E10	328122	BioLegend
NBM	CD38	ECD	LS198-4-3	A99022	Beckman Coulter
NBM/pAML	CLEC12A[§]	PE	HB3	-	-
NBM	CD45RA	FITC	HI100	304106	BioLegend
NBM	CD2	PE-Cy5	RPA2.10	300209	BioLegend
NBM	CD3	PE-Cy5	HIT3α	300309	BioLegend
NBM	CD4	PE-Cy5	RPA-T4	300509	BioLegend
NBM	CD7	PE-Cy5	CD7-6B7	343110	BioLegend
NBM	CD8a	PE-Cy5	RPA-T8	301009	BioLegend
NBM	CD10	PE-Cy5	HI10a	312206	BioLegend
NBM	CD11b	PE-Cy5	ICRF44	301307	BioLegend
NBM	CD14	PE-Cy5	RMO52	A07765	Beckman Coulter
NBM	CD19	PE-Cy5	HIB19	302209	BioLegend
NBM	CD20	PE-Cy5	2H7	302307	BioLegend
NBM	CD56	PE-Cy5	B159	555517	BD Biosciences
NBM	CD235a	PE-Cy5	HIR2	306605	BioLegend
NBM	CD123	PE-Cy7	6H6	306010	BioLegend
NBM	CD34	APC	581	343510	BioLegend
NBM/pAML	Membrane permeability, amine reactive	Zombie NIR	-	423105	BioLegend
pAML	CD38	FITC	HIT2	303504	BioLegend
pAML	CD45	BV510	HI30	563204	BD Biosciences
pAML	CD34	PerCP-Cy5.5	581	343521	BioLegend
pAML	CD3	PB	UCHT1	300431	BioLegend

APC: Allophycocyanin; Bcl-2: B-cell lymphoma 2; BV: Brilliant Violet; CLEC12A: C-type lectin domain family 12 member A; ECD: Electron Coupled Dye, also known as PE-Texas-Red; FITC: Fluorescein isothiocyanate; MoAb: Monoclonal antibody; PB: Pacific Blue; PE: Phycoerythrin; PerCP-Cy5.5: Peridinin-chlorophyll protein-cyanine 5.5; PE-Cy5: Phycoerythrin-Cyanine 5; PE-Cy7: Phycoerythrin-Cyanine7. [§]The CLEC12A monoclonal antibody was produced in-house from the HB3 hybridoma and conjugated by Agilent, Glostrup, Denmark. The leukemia and healthy samples are indicated as pAML and NBM, respectively.
