# Supplementary material for: Proteomic Profiling Identifies Specific Leukemic Stem Cell-Associated Protein Expression Patterns in Pediatric AML Patients
Source: Cancers (Basel). 2022 Jul 22;14(15):3567. doi: 10.3390/cancers14153567 (PMC9332109; doi:10.3390/cancers14153567)
Supplement: Supplementary file 1 [file cancers-14-03567-s001.zip › Table S2_Petersen et al.pdf]

Table S2. Purity of FACS sorted subsets

Cell subset	pAML17		pAML20		pAML21		pAML23		pAML29	
	Events/ events in cell gate	%	Events/ events in cell gate	%	Events/ events in cell gate	%	Events/ events in cell gate	%	Events/ events in cell gate	%
**AML-SC**	51/51	100%	3556/3572	100%	163/163	100%	537/540	99%	701/705	99%
**PC1**	25/27	93%	6655/6734	99%	48/63	76%	1088/1113	98%	856/865	99%
**PC2**	16/16	100%	28/28	100%	95/97	98%	3102/3133	99%	657/659	100%
**BC1**	7054/7071	100%	6013/6053	100%	1615/1641	98%	544/545	100%	652/661	99%
**BC2**	40/43	93%	175/208	84%	612/674	91%	412/425	97%	139/140	99%

Cell subset	NBM09		NBM10		NBM11		NBM12		NBM13	
	Events/ events in cell gate	%	Events/ events in cell gate	%	Events/ events in cell gate	%	Events/ events in cell gate	%	Events/ events in cell gate	%
**HSC**	405/405	100%	n.a.	n.a.	33/35	94%	28/28	100%	12/12	100%
**CMP1**	205/210	98%	51/54	94%	14/18	78%	22/23	96%	23/25	92%
**CMP2**	367/399	92%	82/84	98%	28/31	90%	70/73	96%	28/28	100%
**GMP1**	284/299	95%	41/44	93%	11/12	92%	254/270	94%	53/55	96%
**GMP2**	284/295	96%	336/348	97%	14/14	100%	186/186	100%	24/24	100%
**MEP**	86/86	100%	193/197	98%	18/18	100%	20/23	87%	22/23	96%
**C12A+ Pre**	-	-	132/132	100%	-	-	221/222	100%	70/74	95%

n.a.: not analyzed, due to low cell numbers
